# Metabolomic Perfusate Analysis during Kidney Machine Perfusion: The Pig Provides an Appropriate Model for Human Studies

**DOI:** 10.1371/journal.pone.0114818

**Published:** 2014-12-12

**Authors:** Jay Nath, Alison Guy, Thomas B. Smith, Mark Cobbold, Nicholas G. Inston, James Hodson, Daniel A. Tennant, Christian Ludwig, Andrew R. Ready

**Affiliations:** 1 Department of Renal Surgery, University Hospitals Birmingham, Birmingham, United Kingdom; 2 Department of Immunity & Infection, University of Birmingham, Birmingham, United Kingdom; 3 Wolfson Computer Laboratory, University Hospitals Birmingham, Birmingham, United Kingdom; 4 Henry Wellcome Building for Biomolecular NMR Spectroscopy, University of Birmingham, Birmingham, United Kingdom; 5 School of Cancer Sciences, University of Birmingham, Birmingham, United Kingdom; University Hospital Oldenburg, Germany

## Abstract

**Introduction:**

Hypothermic machine perfusion offers great promise in kidney transplantation and experimental studies are needed to establish the optimal conditions for this to occur. Pig kidneys are considered to be a good model for this purpose and share many properties with human organs. However it is not established whether the metabolism of pig kidneys in such hypothermic hypoxic conditions is comparable to human organs.

**Methods:**

Standard criteria human (n = 12) and porcine (n = 10) kidneys underwent HMP using the LifePort Kidney Transporter 1.0 (Organ Recovery Systems) using KPS-1 solution. Perfusate was sampled at 45 minutes and 4 hours of perfusion and metabolomic analysis performed using 1-D ^1^H-NMR spectroscopy.

**Results:**

There was no inter-species difference in the number of metabolites identified. Of the 30 metabolites analysed, 16 (53.3%) were present in comparable concentrations in the pig and human kidney perfusates. The rate of change of concentration for 3-Hydroxybutyrate was greater for human kidneys (p<0.001). For the other 29 metabolites (96.7%), there was no difference in the rate of change of concentration between pig and human samples.

**Conclusions:**

Whilst there are some differences between pig and human kidneys during HMP they appear to be metabolically similar and the pig seems to be a valid model for human studies.

## Introduction

The use of Hypothermic Machine Perfusion (HMP) in the period between kidney retrieval and implantation is supported by robust clinical evidence with improved early graft outcome [1]–[9]. Flow dynamics during perfusion is likely to account for some of the benefits of HMP, with reduced intra-renal resistance (and therefore increased flow) a marker of good graft function [10]–[12]. However, the exact mechanism by which HMP improves outcome remains unclear and there is likely to be a metabolic component underlying these beneficial effects [13], [14]. Accordingly, improved metabolic support during perfusion becomes a target for graft optimisation. Experimental studies are needed to clarify these mechanisms and optimisation of preservation may lead to improved transplant outcomes, especially in marginal kidneys. However the metabolic activity in this ex vivo, hypoxic, hypothermic environment is poorly understood.

Metabolomic analysis using ^1^H NMR spectroscopy permits identification and quantification of a large number of metabolites within a biological sample and is the subject of great interest. Easy access to perfusate during HMP and ability to perform serial measurements render this an attractive technique with which to establish a reliable biomarker and may even provide the option to improve the metabolic function of organs with obvious potential benefits. We have shown that perfusate analysis of human cadaveric kidneys is feasible and can be used to reliably predict post transplant graft function [15]

Porcine kidneys are a convenient and accessible animal model for experimental studies. They are readily available and have comparable size and physiological properties to human organs [16]–[19]. Within transplantation, porcine models have been studied extensively and normothermic perfusion is a good example of how this has translated into clinical practice [20]. On a functional level, analysis using ^1^H-NMR spectroscopy of perfusate from autotransplanted pig kidneys has demonstrated that metabolite concentrations do correspond to graft outcome indicating that there is a strong correlation between pre-transplant metabolism and graft function [21].

Under normal physiological conditions, the metabolic profiles of porcine blood, kidney tissue, urine and serum have been shown to be comparable to humans [22], [23]. As of yet, this has not been validated in the *ex vivo* hypothermic environment as encountered during HMP. The aim of this study is to compare the metabolic profile of human and porcine kidneys using ^1^H-NMR spectroscopy of HMP-derived perfusate to determine whether the porcine model is a valid surrogate for human studies.

## Methods

### Human subject research

Ethical approval was obtained from the West of Scotland Research Ethics Service for collection of human perfusate samples and subsequent NMR analysis (REC reference number: 12/WS/0166). Written consent for research purposes was granted from donor next of kin and from prospective kidney recipients to allow for collection and publication of postoperative transplant function information.

### Animal Research

Abattoir/slaughterhouse pig kidneys were used in this study, acquired through F.A. Gill, Wolverhampton. No animals were sacrificed solely for the purposes of this study and therefore no ethical board approval was necessary.

### Human studies

Standard criteria (e.g. donor age <60) adult cadaveric kidneys (n = 12) accepted for transplantation and undergoing HMP at the Queen Elizabeth Hospital, Birmingham between July 2012 and August 2013 were included, subject to consent and resource availability. Kidneys from DCD (Donation after Cardiac Death) donors and those fulfilling standard criteria definitions but with predicted delayed graft function (e.g. Cold ischaemia time >16 hours) were excluded to enable valid comparison with the pig group.

Organs were cold stored at 4°C in the period following retrieval according to local retrieval team protocols and transferred to the perfusion machine at the host centre. Decision to preserve organs with HMP was determined by centre policy.

### Pig studies

Experiments were performed on 22–26 week old ‘bacon weight’ pigs, weighing 80–85 kg (n = 10). All experiments were performed following the principles of laboratory animal care according to NIH standards. Animal were sacrificed by electrical stunning and exsanguination. Cold perfusion was performed ex-vivo following laparotomy and retrieval and occurred within 14 minutes of death. Kidneys were initially cold flushed (4°C) with 1L Soltran solution under aseptic conditions at pressure of 150 mmHg. Organs were then cold stored in KPS-1 solution for two hours prior to machine perfusion to replicate human organ cold storage conditions.

### Hypothermic Machine Perfusion

Perfusion pressure for both animal and human organs was set at 30 mmHg and kidneys were perfused with 1 L of KPS-1 at 4°C using LifePort Kidney Transporter 1.0 (Organ Recovery Systems). Separate devices were used for human and animal studies. 2 mL of perfusate was sampled at 45 minutes and 4 hours for each kidney. Perfusate was transferred to a cryogenic vial and stored at −20°C until thawed at room temperature, prepared and processed.

### Sample preparation

NMR samples were prepared by mixing 150 µL of 400 mM (pH 7.0) phosphate buffer containing 2 mM TMSP [(3-trimethylsilyl)propionic-(2,2,3,3-d4)-acid sodium salt] with 390 µL of each perfusate sample and 60 µL of deuterium oxide (D_2_O) to reach a final phosphate buffer concentration of 100 mM and a final TMSP concentration of 500 µM. After mixing, the 600 µL samples were pipetted into NMR tubes and centrifuged to remove any air bubbles.

^1^H-NMR spectra were acquired using a Bruker AVII 500 MHz spectrometer equipped with a 5 mm inverse Cryoprobe. The sample temperature was set to 300 K, excitation sculpting was used to suppress the water resonance [24]. One-dimensional spectra were acquired using a 6 kHz spectral width, 32768 data points, 4 s relaxation delay and 128 transients. Matching was manual prior to acquisition of first sample and each sample was automatically shimmed (1D-TopShim) to a TMSP line width of less than 1 Hz prior to acquisition. Samples with a TMSP line width >1 Hz were acquired again after manual shimming where the TMSP half height line width was shimmed below 1 Hz. Total experimental time was approximately 15 minutes per sample.

All data sets were processed using the MATLAB based MetaboLab software [25]. Data sets were zero filled to 65536 data points. An exponential line broadening of 0.3 Hz was applied before Fourier transformation. The chemical shift axis was calibrated by referencing the TMSP signal to 0 ppm. Spectra were manually phase corrected and baseline correction using a spline before segmental alignment of all resonances using Icoshift [26]. Spectra were then exported into Bruker format.

**Figure 1:**
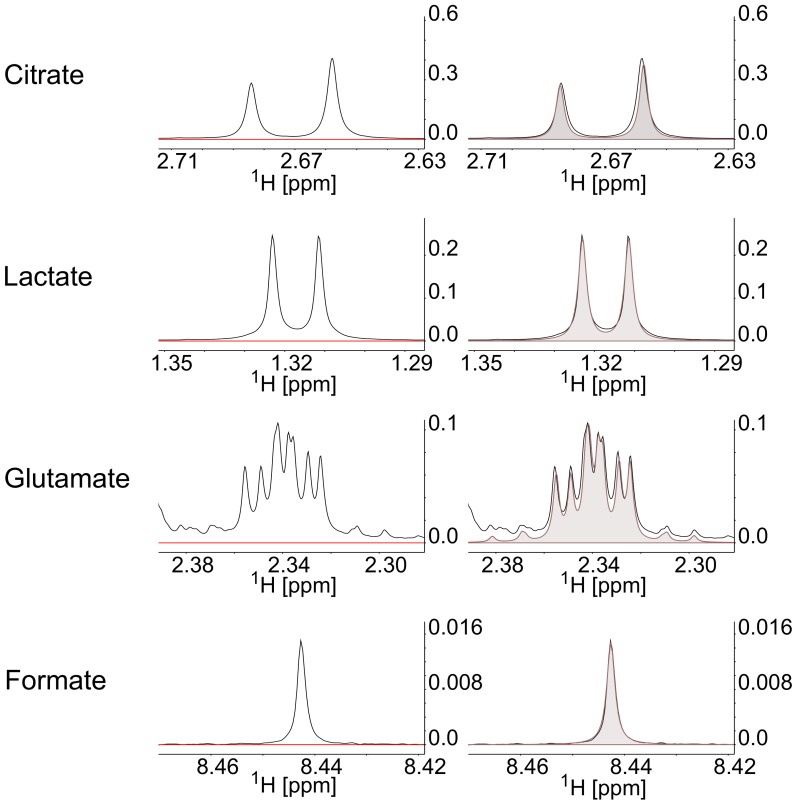
Example metabolic quantification using Chenomx database. Localised spectral plots for metabolites of interest with shaded figures illustrating metabolite quantification via best fit analysis using Chenomx metabolite database.

Resultant spectra were examined using Chenomx 7.0 (ChenomxInc) profiling to identify metabolites and their concentrations, as illustrated in Fig. 1. Concentrations were corrected to compensate for the dilutional effect of the buffer.

### Statistical Methodology

Prior to analysis, the distribution of metabolites was examined. Where non-normality was detected, Log_10_-transformations were applied, after adding 1 to remove zero values. Repeated measures ANOVA models were then used to compare metabolite concentrations, both between pig and human samples, and between 45 minute and 4 hour timepoints. In addition to the main effect terms in the models, interactions were also included, in order to compare the rate of change over time in the metabolite concentrations between pig and human samples.

Data were reported as arithmetic means and 95% confidence intervals for the normally-distributed data. Where Log-transformations were used, the resulting summary statistics were back transformed, and reported as geometric means and 95% confidence intervals.

All analysis was performed using IBM SPSS 19 (IBM Corp. Armonk, NY), with p<0.05 deemed to be indicative of statistical significance.

## Results

Metabolic support may be an important factor in the observed benefit of HMP compared with cold storage for preserving kidneys prior to transplantation. Experimental studies are needed to detail the metabolic activity of kidneys under various storage conditions, but the usage of healthy human kidneys for such research purposes is not justified.

This study seeks to ascertain whether the abundant and accessible porcine kidney can provide a reliable metabolic model for the human kidney during HMP.

Metabolite concentrations as determined from ^1^H-NMR spectra from 10 porcine kidneys were compared with 12 standard criteria cadaveric human kidneys, all of which were successfully transplanted with immediate graft function.

**Figure 2:**
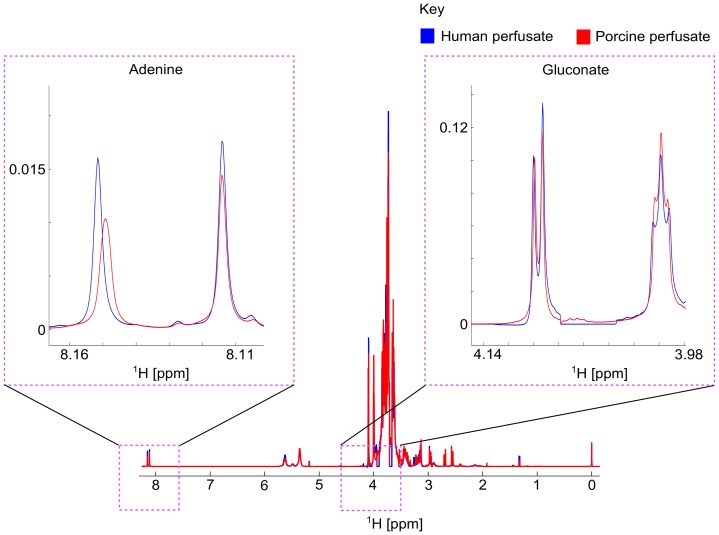
^1^H-NMR spectral overlay plot demonstrating the similarity of HMP perfused pig (red) and human (blue) kidneys after 4 hours of perfusion.

A spectral overlay analysis was performed for the mean spectra for pig and human samples at 45 minutes and 4 hours. There were similar profiles for both pig and human groups (Fig. 2). There were no metabolites consistently present in significant quantities that were not detected in the other group.

In total, 30 metabolites were identified in the perfusate of both pig and human kidneys during hypothermic machine perfusion. Of these, 6 (gluconate, mannitol, glucose, adenine, ribose and glutathione) were constituents of the original KPS-1 perfusion fluid. There was consumption of glutathione in both pig and human groups but no other significant interspecies or time effect differences for the other five metabolites present.

For the 24 metabolites present *de novo* (therefore likely produced by the kidney), there was an overall change over time for 12, with production of lactate, glycine, glutamate, hypoxanthine, alanine, 3-hydroxybutryate, inosine, N-phenylacetylglycine, leucine, valine, isoleucine and fumarate.

**Figure 3:**
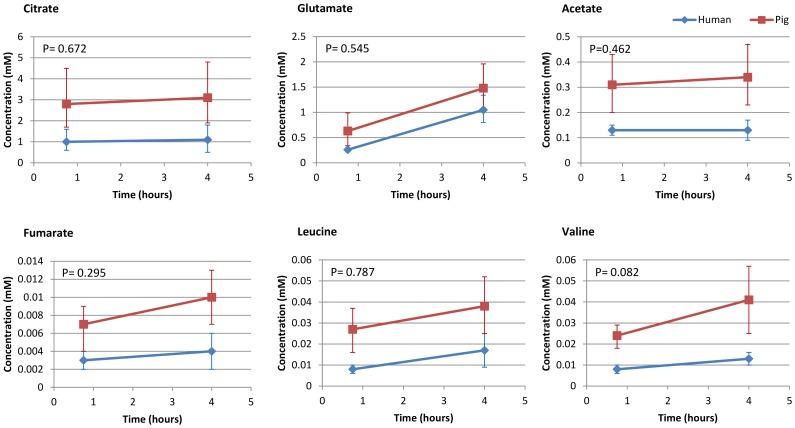
Comparable rate of change of concentration demonstrated for depicted metabolites during 4 hours of HMP despite absolute interspecies concentration differences.

When concentrations were analysed according to species, there was no difference during HMP between pig and human kidneys for 16 metabolites as assessed using a repeated measures analysis. The rate of change of concentration for 3-hydroxybutyrate was greater in human kidneys compared to pig kidneys (0.017 to 0.040 mM vs 0.012 to 0.013 mM) (p<0.001). The vast majority of metabolites detected (29/30) demonstrated no difference in the rate of change between pig and human samples (Table 1, Fig. 3).

**Table 1:** Concentration of metabolites present after 45 minutes and 4 hours of perfusion in pig and human kidneys.

		Time point		p-Values		
	Species	*45 Minutes*	*4 Hours*	*Time*	*Species*	*Int.*
***Gluconate***	Human	92.9 (85.3–100.4)	96.6 (84.1–109.2)	0.799	0.342	0.616
	Pig	89.6 (78.4–100.7)	88.3 (76.4–100.3)			
***Mannitol***	Human	48.8 (45.6–52.1)	52.3 (46.0–58.5)	0.543	0.368	0.667
	Pig	53.3 (45.8–60.7)	53.9 (45.0–62.7)			
***Glucose***	Human	9.8 (9.0–10.6)	10.7 (9.6–11.7)	0.158	0.088	0.709
	Pig	11.4 (9.3–13.5)	12.9 (9.7–16.1)			
***Adenine***	Human	7.0 (5.8–8.1)	7.1 (5.7–8.5)	0.924	0.681	0.816
	Pig	6.7 (5.7–7.7)	6.7 (5.4–7.9)			
***Ribose***	Human	3.0 (2.8–3.3)	3.0 (2.4–3.6)	0.548	0.147	0.718
	Pig	3.7 (2.9–4.5)	3.4 (2.6–4.3)			
***Glutathione***	Human	1.3 (1.2–1.4)	0.8 (0.6–0.9)	<0.001*	0.731	0.1
	Pig	1.4 (1.2–1.7)	0.6 (0.4–0.8)			
***Malonate***	Human	2.36 (2.04–2.67)	2.42 (1.90–2.95)	0.778	0.855	0.91
	Pig	2.26 (1.26–3.26)	2.41 (1.58–3.23)			
***Citrate*** #	Human	1.0 (0.6–1.6)	1.1 (0.5–1.8)	0.478	0.005*	0.672
	Pig	2.8 (1.7–4.5)	3.1 (1.9–4.8)			
***Lactate*** #	Human	0.94 (0.80–1.09)	1.88 (1.49–2.33)	0.002*	0.005*	0.057
	Pig	0.73 (0.37–1.17)	0.93 (0.69–1.22)			
***Glycine*** #	Human	0.58 (0.47–0.70)	1.86 (1.40–2.41)	<0.001*	0.086	0.683
	Pig	0.87 (0.67–1.09)	2.20 (1.65–2.85)			
***Glutamate*** #	Human	0.26 (0.22–0.30)	1.05 (0.80–1.34)	<0.001*	0.013*	0.545
	Pig	0.63 (0.34–0.99)	1.48 (1.07–1.96)			
***Hypoxanthine*** #	Human	0.17 (0.12–0.22)	0.29 (0.22–0.36)	0.005*	0.782	0.888
	Pig	0.19 (0.10–0.28)	0.30 (0.16–0.45)			
***Acetate*** #	Human	0.13 (0.11–0.15)	0.13 (0.09–0.17)	0.507	<0.001*	0.462
	Pig	0.31 (0.20–0.43)	0.34 (0.23–0.47)			
***Formate*** #	Human	0.10 (0.07–0.13)	0.11 (0.08–0.15)	0.594	0.306	0.411
	Pig	0.13 (0.09–0.18)	0.13 (0.09–0.18)			
***Alanine*** #	Human	0.08 (0.07–0.09)	0.20 (0.16–0.24)	<0.001*	0.961	0.133
	Pig	0.10 (0.08–0.12)	0.18 (0.13–0.23)			
***Creatinine*** #	Human	0.031 (0.020–0.043)	0.057 (0.049–0.065)	0.084	0.031*	0.558
	Pig	0.080 (0.029–0.133)	0.133 (0.040–0.233)			
***Ethanol*** #	Human	0.024 (0.021–0.027)	0.036 (0.010–0.063)	0.385	0.018*	0.573
	Pig	0.076 (0.040–0.114)	0.079 (0.037–0.123)			
***Isopropanol*** #	Human	0.023 (0.017–0.030)	0.025 (0.018–0.032)	0.792	0.063	0.167
	Pig	0.017 (0.015–0.019)	0.016 (0.014–0.018)			
***3-Methylxanthine***	Human	0.020 (0.016–0.025)	0.024 (0.019–0.028)	0.361	0.118	0.44
	Pig	0.017 (0.010–0.023)	0.017 (0.010–0.023)			
***3-Hydroxybutyrate***	Human	0.017 (0.013–0.021)	0.040 (0.031–0.048)	<0.001*	<0.001*	<0.001*
	Pig	0.012 (0.006–0.017)	0.013 (0.005–0.020)			
***Inosine*** #	Human	0.017 (0.008–0.025)	0.023 (0.011–0.035)	0.038*	0.008*	0.241
	Pig	0.003 (0.001–0.004)	0.005 (0.002–0.007)			
***Uracil***	Human	0.011 (0.010–0.012)	0.018 (0.013–0.023)	0.05	0.020*	0.258
	Pig	0.007 (0.005–0.008)	0.009 (0.000–0.017)			
***N-Phenylacetyl glycine*** #	Human	0.011 (0.005–0.016)	0.022 (0.010–0.034)	0.041*	0.18	0.094
	Pig	0.009 (0.003–0.014)	0.010 (0.004–0.016)			
***Pyruvate*** #	Human	0.011 (0.006–0.016)	0.010 (0.005–0.015)	0.371	0.112	0.24
	Pig	0.012 (0.005–0.019)	0.020 (0.010–0.030)			
***Leucine*** #	Human	0.008 (0.006–0.010)	0.017 (0.009–0.025)	0.020*	<0.001*	0.787
	Pig	0.027 (0.016–0.037)	0.038 (0.025–0.052)			
***Valine*** #	Human	0.008 (0.006–0.010)	0.013 (0.010–0.016)	0.002*	<0.001*	0.082
	Pig	0.024 (0.018–0.029)	0.041 (0.025–0.057)			
***Tyrosine*** #	Human	0.007 (0.001–0.012)	0.006 (0.005–0.008)	0.132	0.001*	0.083
	Pig	0.014 (0.010–0.017)	0.020 (0.014–0.026)			
***Hippurate*** #	Human	0.005 (0.002–0.007)	0.027 (0.000–0.067)	0.298	0.198	0.337
	Pig	0.001 (0.000–0.002)	0.002 (0.000–0.004)			
***Isoleucine*** #	Human	0.004 (0.004–0.005)	0.008 (0.006–0.010)	0.001*	<0.001*	0.074
	Pig	0.014 (0.010–0.017)	0.024 (0.016–0.032)			
***Fumarate*** #	Human	0.003 (0.002–0.004)	0.004 (0.002–0.006)	0.012*	0.002*	0.295
	Pig	0.007 (0.004–0.009)	0.010 (0.007–0.013)			

Data reported as “Arithmetic Mean (95% Confidence Interval), unless stated otherwise.

# Analyses were log-transformed in the analysis, hence are reported as “Geometric mean (95% CI).

p-values: Time – Main effect of measurement time; Species – Main effect of species; Int. – Interaction between measurement time and species.

*Significant at p<0.05.

## Discussion

The aim of this study was to determine whether or not the porcine kidney provides a reliable metabolic model for the human kidney, as assessed by ^1^H-NMR.

Whilst it is recognized that ^1^H-NMR can be used to detect metabolic changes present within the perfusates during HMP, this paper demonstrates that in both human and pig studies a significant amount of metabolic activity occurs. Some interspecies metabolite differences are evident but nevertheless, the similarity between the two groups is striking. This appears to validate porcine HMP as a valid metabolic model for human studies and would suggest that a defined optimal metabolic support protocol for HMP in a pig model would be translatable into clinical practice.

The majority of metabolites were present in similar concentrations in both species. For metabolites where concentration differences were observed, all but 3-hydroxybutryate had comparable rates of change in concentration for pig and human samples. This would imply that the active metabolic pathways during HMP in both human and pig kidneys are broadly comparable. The kidney cannot synthesise the ketone body 3-hydroxybutyrate to any significant extent but can consume it as an energy source and is likely to be more pronounced in stressful conditions such as hypothermia [27], [28]. Interspecies differences in the levels of the enzyme responsible for metabolising 3-hydroxybutyrate, 3-hydroxybutyrate dehydrogenase within the renal cortex and in plasma levels of 3-hydroxybutyrate have been reported and may account for this finding [29].

The pig kidneys were subjected to a warm ischaemia time of 14 minutes which is more prolonged than many Donation after Brain Death (DBD) human kidneys. However the cold ischaemic time for the pig organs (2 hours) was shorter than the human group in this study (mean 8 hr 40 min). There was also a significant age discrepancy between the pig and human organs, with the older human kidneys also likely subject to the global metabolic changes associated with brain death prior to retrieval including thyroid, catecholamine and glycaemic effects [30]–[35]. Such difference in retrieval conditions is likely to account for many of the interspecies differences found such as the trend towards higher levels of lactate in the human kidneys.

The authors acknowledge that the good quality standard criteria human kidneys in this study are not representative of many of the organs used in clinical practice. Indeed extended criteria organs may have most to gain from metabolic optimistion during machine perfusion and further studies would be of value. Whilst the number and concentration change of the metabolites in pig and human perfusates are comparable in this study, it lacks the power to detect small interspecies differences. Furthermore in 1-D spectra, metabolites with small single peaks can be obscured by more dominant signals from other metabolites with similar chemical shifts.

Perfusate analysis determines the concentration of metabolites in the extracellular environment of the kidney. Whilst the intracellular activity of many metabolites can be inferred from this, metabolomic analysis of kidney tissue would provide a more detailed account of the intracellular milieu and is a limitation of this study.

This study demonstrates that ^1^H-NMR spectroscopy profiles of perfusate samples for porcine and human kidneys during HMP are similar and implies that similar metabolic processes occur during preservation in the two species. This further validates the pig as a model for human transplantation and for HMP in particular.
